# Extracellular Vesicles: New Perspectives of Regenerative and Reproductive Veterinary Medicine

**DOI:** 10.3389/fvets.2020.594044

**Published:** 2020-11-16

**Authors:** Elena Yu Zakirova, Alexande M. Aimaletdinov, Albina G. Malanyeva, Catrin S. Rutland, Albert A. Rizvanov

**Affiliations:** ^1^Institute of Fundamental Medicine and Biology, Kazan Federal University, Kazan, Russia; ^2^Faculty of Medicine and Health Sciences, School of Veterinary Medicine and Science, University of Nottingham, Nottingham, United Kingdom

**Keywords:** extracellular vesicles, personalized medcine, stem cell, regenerative veterinary medicine, reproductive veterinary medicine

## Abstract

Extracellular vesicles are released by all cell types including stem cells. Stem cell-released extracellular vesicles have therapeutic effects similar to those of their parent cells and have regenerative effects in tissues. They also have an immunomodulating effect when down-regulating some proinflammatory factors, without exerting effects on cell proliferation, modulating angiogenesis or altering cellular functions in recipient cells. Modern veterinary research explores vesicles and creates or advances methods of using them in regenerative and reproductive medicine, applications of these technologies are under development.

## Introduction

Extracellular vesicles are nano-sized cell membrane particles released by different cells and isolated from most biological fluids. Vesicles play a central role in intercellular communications as they contain proteins, various RNAs, DNAs, lipids, and other biomolecules which are targeted for intercellular delivery and exchange (paracrine signaling) both at system and local tissue levels. Extracellular vesicles also regulate normal physiological processes in organisms (1). They are a heterogeneous group of cell-derived membranous structures comprised of exosomes, microvesicles (MV) and apoptotic bodies which are released under physiological conditions as well as in cell activation, aging and apoptosis (Table 1). According to the literature their release can also maintain cell integrity by eliminating harmful substances from a cell (2). Moreover, Badierah et al. noticed that extracellular vesicles can spread viral infection in the body overcoming the immune system (3).

**Table 1:** Classification of extracellular vesicles.

**Extracellular vesicles**	**Size, nm**	**Formation**	**Internal content**	**Supposed functions**
Aoptotic bodies	1,000–5,000	Cell defragmentation into parts, surrounded by a cellular membrane	Genome DNA, whole organelles, rRNA	Phagocytosed by cells, a transport function is possible
Microvesicles	100–1,000	Cellular membrane protrusion and pinching into extracellular space	Parent cell markers, enzymes, transcription factors, molecules of mRNA	Mediate a distant interaction between cells
Exosomes	30–150	Budded into a cell cavity, forming an ednosome which fuses with a surface cell membrane and an aggregation of exosomes is extruded/forced out	A particular set of membrane and cytosol proteins, mRNA, siRNA	Mediate a distant interaction between cells

*DNA, deoxyribonucleic acid; RNA, ribonucleic acid; mRNA, matrix ribonucleic acid; siRNA, small interfering ribonucleic acid; rRNA, ribosomal ribonucleic acid*.

Stem cells (SCs) are known to be capable of producing extracellular vesicles. SC-released extracellular vesicles with a paracrine mechanism exert a therapeutic effect similar to that of their parent cells (4). At present, the research of properties and possible ways to use these SC derivatives is a promising trend in biology and medicine (5) as well as the use of artificial MV (6). However, this is understudied in veterinary medicine (7).

## *In vitro* Study of Biological Properties of Animal Extracellular Vesicles

**Figure 1:**
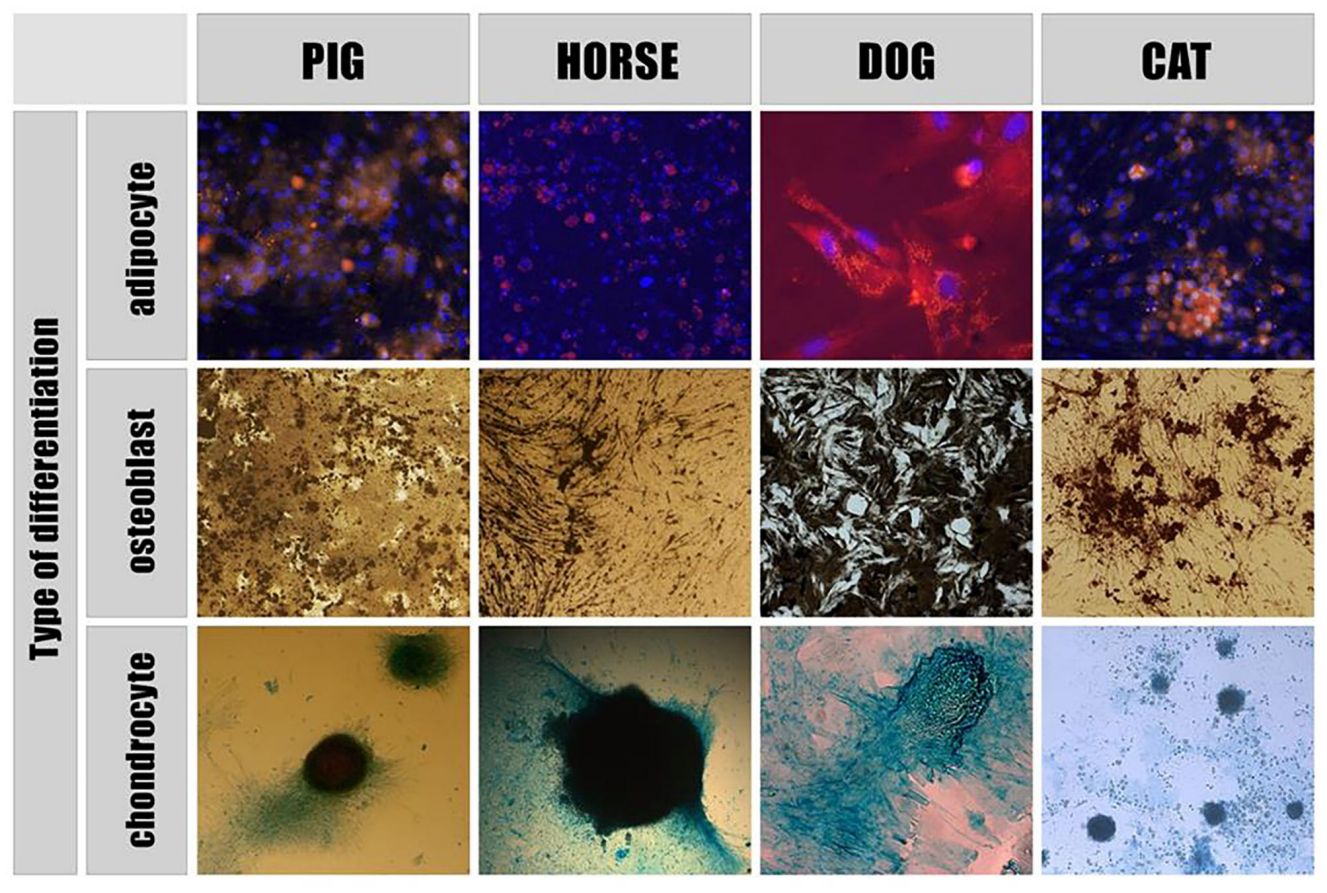
Directed differentiation of adipose - derived stem cells. Adipogenic differentiation: MSCs nuclei are stained with Dapi (blue), adipose tissue inclusions within cells are dyed with Nile Red (yellow). Osteogenic differentiation: Von Kossa staining showed calcium containing areas as brown in color. Chondrogenic differentiation: acid mucopolysaccharides of the extracellular matrix formed within the differentiation of MSCs into chondrocytes are stained with Alcian blue.

According to the literature, extracellular vesicles have presently been derived and characterized from porcine, canine and equine mesenchymal stem cells (MSCs) as well as from equine amnion mesenchymal stromal cells (aMSCs). The Mesenchymal and Stem Cells Committee of the International Society for Cellular Therapy stated minimal criteria for defining MSCs. They must be plastic-adherent, have a specific surface membrane markers profile and be able to differentiate into osteoblasts, adipocytes and chondrocytes *in vitro* (Figure 1).

Immunophenotyping is one of the most relevant tests to define cells derived as MSCs. For example, they must express markers such as CD105, CD73, CD29, CD44, CD106, CD166, CD90, but lack the expression of CD45, CD34, CD14, CD11b, CD79a, CD19 (8, 9).

Two main sources of MSCs such as bone marrow (BM) and adipose tissue (AT) are usually used for the treatment of dogs in regenerative veterinary medicine. Investigators note that despite a similar immunophenotype manifested by BM- and AT-MSCs, AT-MSCs act on cell proliferation more actively than BM-MSCs do while the latter have a significantly higher secretory production of some soluble factors, as well as exosomes under standard culturing conditions. Thus, it is recommended that biological differences are taken into consideration when selecting a source of MSCs for cell transplantation (10).

Studies demonstrated that equine AT-MSCs were able to produce extracellular vesicles. Horse MSC-derived membrane vesicles could have an effect during a key stage in tissue regeneration, angiogenesis (11, 12). The further study of equine AT-MSC derivatives showed that an extracellular vesicle membrane contained CD90 and flotillin-1, which are considered as markers of MV and exosomes, respectively (13); therefore, equine AT-MSCs express extracellular vesicles of two types. In general, membrane vesicles of equine MSCs could potentially be used as a therapeutic tool in regenerative veterinary medicine.

Extracellular MVs of aMSCs in an *in vitro* model of horse tendinitis have an immunomodulating effect when down-regulating some proinflammatory factors without any effect on cell proliferation (14). It was shown that these effects were due to microvesicle miRNAs. For example, miRNA-150 reduces the secretion of inflammatory cytokines (15). Protective properties identified in extracellular MVs would facilitate development of the principle of using these cell derivatives in a veterinary practice for the treatment of tendinitis for example, as tendon and ligament injuries in sporting horses. At present regeneration of damaged tissue is challenging. This is due to the limited abilities of tendon and ligament tissue regeneration primarily due to scarring as a result of a relatively low number of resident cells as compared to the matrix (16).

Pig AT-MSC-derived extracellular vesicles contain mRNAs of transcription factors and genes involved in angiogenesis and adipogenesis, as well as those of the Golgi complex. At the same time, mitochondrial, calcium signal, and cytoskeletal genes are selectively removed from them. The analysis of pig AT-MSC-derived extracellular vesicles demonstrates that their miRNAs regulate functions of transcription factors and genes which are involved in some cellular processes including angiogenesis, cellular transport, apoptosis, and proteolysis. These cell derivatives transport regulatory information to modulate angiogenesis, adipogenesis, and other cellular functions in recipient cells. The results described may assist in developing regenerative strategies for the treatment of various diseases in animals with the use of extracellular vesicles (17).

## Potential Use of Extracellular Vesicles in Veterinary Medicine

Sabry et al. conducted an experimental study of dog BM-MSC-derived microvesicle effects on the regeneration of an induced chondral defect. Chondral defects were created surgically in canine femoral condyles. The investigators isolated autologous MSCs and obtained natural MVs when culturing *in vitro*, which were infra-articularly injected. A saline solution was injected into another injured joint as a negative control. MVs obtained in culturing dog BM-MSCs exerted an evident proliferative effect on dog chondrocytes in an *in vitro* experiment. In *in vivo* studies where MVs were injected into dog joints, the cartilage evidently regenerated and the chondral histomorphology restored in the experimental group at the end of a 6-month follow-up period as compared to control joints in which the condition got worse with the time and the defect was filled with fibrous tissue forming a fibrous cartilage. Researchers concluded that autologous dog BM-MSC derived MVs were effective for the restoration of the injured cartilage functions and morphology. This approach can be used as the concept of cell-free therapy in regenerative and veterinary medicine (18).

Natural MVs obtained when culturing dog BM-MSCs were also effective for regeneration of full-thickness skin following wo unds in dogs. The histological findings in the experimental group showed that the use of MVs stimulated skin wound healing, even on day 3 after their use, and collagen synthesis. This not only promoted the formation of new blood vessels but also accelerated vascular development and maturation. Therefore, the use of MVs for the treatment of skin wounds is promising in relation to the development of a novel cell-free therapeutic approach which might overcome risks related to SC transplantation (19).

## Use of Extracellular Vesicles in Veterinary Reproductive Conditions

The conservation of genetic material from animals with a high breeding value or those endangered as a result of any disease or a disaster is one of the current issues in veterinary science and medicine. This is achieved by freezing mammal semen and oocytes.

The use of cryoconserved sperm in dogs reduces problems accompanying natural breeding, transportation and international trading. However, the process of freezing can have a particularly adverse impact on the morphology of spermatozoon caused by thermal, mechanical, chemical, osmotic, and oxidative damage (20). These effects result in the reduction in spermatozoon motility, DNA damage. Overall these result in a reduction in spermatozoon fertilizing capacity. The restoration of spermatozoon impaired by freezing is extremely important for the improvement of its viability and fertility. Researchers studied the effects of canine AT-MSC-derived exosomes on dog spermatozoon structure and functions following cryoconservation. Having been thawed the spermatozoon previously treated with exosomes had better motility, a higher percentage of living sperm, and better membrane and acrosome integrity as compared to the controls. Moreover, the expression of genes associated with plasmalemma and chromatin material reparation was statistically higher in exosome-treated sperm than in the controls; however, the expression of a mitochondrial modulator gene of reactive oxygen species 1 was significantly higher in the controls (20).

There are data available on the effects of MVs secreted by multicellular spheroids of cells isolated from dog oviducts have on (dog) oocyte maturation. Results obtained suggest that MVs in specific concentrations had a positive effect on the oocyte maturation rate. They also showed effects on target cells, even without a direct contact between cells, that is exampled by oocytes and the secretome produced by dog oviduct cells. The investigators considered that the maintenance of oocyte viability and maturation was affected by specific miRNAs in substances dissolved in MVs rather than these substances alone. This study clarifies a mechanism of possible paracrine effects the oviduct has on oocytes and is significant for the development of *in vitro* biotechnologies in veterinary medicine (21).

Ferraz et al. found that the majority of extracellular vesicles which are present in the domestic cat oviduct are exosomes. Experiments *in vitro* demonstrated that incubation of cat spermatozoon with cat oviduct exosomes maintained a higher percentage of motile spermatozoon as compared to control intact ones, and persisted for a 24-h. Therefore, a spermatozoon receives energy from molecules which are present in a spermatic fluid and in female reproductive organs. Increased motility might have been due to the transfer of proteins, related to energy metabolism from exosomes into spermatozoa. In addition, there was an increased fertilizing ability that seems to be caused by proteins, related to recognition and binding of sperm cells with oocytes. *In vitro* studies are of great importance for captive breeding of endangered cats (and many other species) as a result of them losing natural habitats. Adjuvant reproductive technologies including artificial fecundation and *in vitro* fertilization used to maintain genetic diversity and to prevent in-bred depression (22).

Equine aMSC MVs in a model of horse endometriosis exert a prominent anti-inflammatory effect. It is supposed that miRNAs in aMSC MVs stimulate cell proliferation, reduce the expression of pro-inflammatory genes and decrease secretion of pro-inflammatory cytokines *in vitro*. The results obtained are of practical value as endometritis is the main cause of decreased fertility, conception rate, prolongs periods from calving to conception, and results in culling and ultimately in economic and food production losses too, in mares and cattle which occurs in artificial fecundation (15).

At present the use of extracellular vesicles as a potential cell-free therapy in veterinary medicine is promising for the treatment of different animal diseases and is a novel therapeutic strategy. However, a limited yield and labor-consuming procedures to isolate natural extracellular vesicles make large-scale pharmaceutical production challenging and presently restricts their wider use in pre-clinical and clinical trials. There are modern developments which can support these aims, such as release of extracellular vesicles which is stimulated with cytochalazine B. The use of cytochalasin B-induced membrane vesicles as vectors for the delivery of nanoparticles and drugs, when reducing *in vivo* toxicity of chemotherapy in medical studies has been reported (23, 24). However, these studies have not been conducted in veterinary fields. Nevertheless, a similar technique to obtain extracellular vesicles is applicable in veterinary medicine and makes it possible to develop new methods for the regeneration of damaged tissue and the maintenance of animal reproductive health.

## Author Contributions

EZ and AA conceived of the presented idea. EZ wrote the manuscript with support from AA, AM, CR, and AR supervised the project. All authors discussed the results and contributed to the final manuscript and provided critical feedback and helped shape the research, and analysis and manuscript.

## Conflict of Interest

The authors declare that the research was conducted in the absence of any commercial or financial relationships that could be construed as a potential conflict of interest.
